# Utility of biomarkers in predicting complications and in-hospital mortality in patients with COVID-19

**DOI:** 10.12669/pjms.38.5.5165

**Published:** 2022

**Authors:** Noman Ali, Nazir Najeeb Kapadia, Dure Aymen, Noor Baig

**Affiliations:** 1 Noman Ali, Department of Emergency Medicine, Aga Khan University Hospital, Stadium Road, Karachi, Pakistan; 2 Nazir Najeeb Kapadia, MBBS, FCPS (EM) Instructor, Department of Emergency Medicine, Aga Khan University Hospital, Stadium Road, Karachi, Pakistan; 3 Dure Aymen, MBBS Resident, Department of Emergency Medicine, Aga Khan University Hospital, Stadium Road, Karachi, Pakistan; 4 Noor Baig, MBBS, FCPS (Internal Medicine) Assistant Professor, Department of Emergency Medicine, Aga Khan University Hospital, Stadium Road, Karachi, Pakistan

**Keywords:** Biomarkers, Complications, Mortality, Emergency Department

## Abstract

**Objectives:**

To determine the association between the laboratory biomarkers (C-reactive protein (CRP), Ferritin, lactate dehydrogenase (LDH), Procalcitonin, and D-dimer) with complications and in-hospital mortality in COVID-19 patients.

**Methods:**

This single-center, cross-sectional study was conducted at the Department of Emergency Medicine of Aga Khan University Hospital from April 01, 2020, to July 31, 2020. Descriptive statistics were presented as Mean±SD and Median along with Range. The frequencies and percentages were calculated for all categorical variables. Univariate and multivariate analysis was carried out to evaluate the significant association between the laboratory biomarkers and in-hospital mortality.

**Results:**

A total of 310 adult COVID positive patients were included. The most common complication was acute respiratory distress syndrome (ARDS) (37.1%), followed by myocardial injury (MI) (10.7%), deep vein thrombosis (DVT) (0.6%), and pulmonary embolism (PE) (0.3%). In-hospital mortality was 15.2%. In univariate analysis, it was observed that increased values of all biomarkers were significantly associated with the prediction of in-hospital mortality using binary logistic regression analysis (OR > 1.0, P <0.05). In multivariate analysis, increased levels of LDH and D-dimer at admission were significantly associated with increased odds of mortality (P <0.05).

**Conclusion:**

Serum CRP, ferritin, Procalcitonin, LDH, and D-dimer levels at the time of admission can predict complications like ARDS and MI and also predict mortality in COVID-19 infection. Serum LDH and D-dimer are the best amongst them for predicting mortality.

## INTRODUCTION

Coronavirus disease-19 (COVID-19) is a highly contagious infectious disease which is caused by severe acute respiratory syndrome coronavirus 2 (SARS-CoV-2). The virus belongs to the Coronaviridae family and was previously known as the 2019 novel coronavirus (2019-nCoV). Initially, the spread started from a province of China, Wuhan and cases began to rise in late December 2019.^1^ The current outbreak was officially recognized as a pandemic in March 2020. The disease has taken over more than 200 countries under its effect.^2^

COVID-19 has a broad spectrum of clinical manifestations that can range from asymptomatic disease to septic shock and multiorgan failure. Mild disease may mimic the symptoms of an upper respiratory tract infection whereas patients with severe infection can be critically sick at the time of their initial presentation and may need mechanical ventilation support because of acute respiratory distress syndrome (ARDS) and septic shock.^3^,^4^

As the pandemic has stretched the healthcare systems globally, it is important to identify the factors that can help to predict complications and patients’ outcomes. Laboratory biomarkers are surrogate parameters that reflect the pathophysiology of the disease and assist clinicians in early recognition of the severity, complications, and outcomes thus allowing for the appropriate and adequate provision of healthcare resources.^5^

A reasonable number of studies have already been done on the factors that predict severity and prognosis in COVID-19 patients. Multiple studies have proven that severe cases of COVID-19 disease are associated with elevated levels of various biomarkers like C-reactive protein (CRP), Ferritin, interleukin-6, as compared to milder cases in which survival was the outcome.^6^,^7^ Although the literature has provided us initial evidence about the role of laboratory biomarkers in SARS-CoV-2 infection, these findings cannot be generalized due to limitations of small cohorts.

Based on these recent data and clinical evidence, our clinicians in Pakistan have now been measuring a variety of laboratory biomarkers in COVID-19 patients but the clinical impact of these biomarkers on outcomes in our population is still not well defined. In this study, we aimed to determine the association between these biomarkers with complications and in-hospital mortality in COVID-19 patients presented to the Emergency Department (ED).

## METHODS

This single-center, cross-sectional study was conducted at the Department of Emergency Medicine of the Aga Khan University Hospital from April 01, 2020, to July 31, 2020. We proceeded with the study after obtaining ethical approval from the Ethical Review Committee of the Aga Khan University Hospital on June 18, 2020 (ERC # 2020-4994-10746).

All adult COVID positive patients (18 years and above) presenting to the ED during the study period in whom all biomarkers were performed were included in the study. A structured proforma was drafted to collect the following information through medical files and electronic health records i.e., patient demographics, clinical data (signs and symptoms, severity of disease), investigation performed, patient’s disposition, complications occurred, length of stay, and outcomes. Upon admission, in addition to routine baseline laboratory tests, CRP, Ferritin, D-dimer, lactate dehydrogenase (LDH), and procalcitonin (PCT) were also recorded

Data was collected by our two senior residents. Filters such as fever, shortness of breath, cough, and COVID-19 were applied to extract the medical record numbers from triage data. Files were reviewed by the data collectors. Before the initiation of data collection, the data collectors went through a refresher training session to understand the process of Hospital Information Management System to extract files from the medical record and to review the patient file, and extract relevant information based on the questionnaire.

The data entry and analysis were done by using IBM SPSS statistical package for windows version 20 & R programming software using R-studio version 1.2.4. Descriptive statistics were presented as Mean±SD and Median along with Range.

Mean and standard deviation was calculated for the continuous variables whereas the frequencies and percentages were calculated for the categorical variables. Mann Whitney U test was applied to compare the median values along with an interquartile range of all biomarkers difference with complications like ARDS, myocardial injury (MI), deep vein thrombosis (DVT), and pulmonary embolism (PE). All biomarker variables were also centered and scaled. Calculating discriminated power of all biomarkers by threshold value using Receiver operating characteristic (ROC) curve and area under curve (AUC) were used to analyze the optimal cut-off for prediction of mortality. The optimal threshold was also determined by using Youden index criteria to pick the sensitivity and specificity and likelihood ratio of all biomarkers. Univariate and multivariate analysis was carried out to evaluate the significant association between the laboratory biomarkers and in-hospital mortality. Binary logistic regression analysis was applied by taking prediction of mortality (Survival vs. Non-Survival) as a dependent variable and other biomarker variables taking experimental or predictor variable. In the prediction of mortality, the category of non-survival was taken as a reference or control category. All Results were presented in tables and graphs. All tests were applied assuming a 95% confidence level, considering a statistically significant value when p <0.05

## RESULTS

A total of 310 patients were included in the study. The mean age was 57.5+14.2 years. Most of the patients belonged to the male gender category (72.6%). Mild, moderate, and severe to critically cases accounted for 18.4%, 29.7%, and 51.9% respectively. Complications occurred in 130 patients (41.9%). The most common complication was ARDS (37.1%), followed by MI (10.7%), DVT (0.6%), and PE (0.3%). Most of the patients got discharged (84.8%). In-hospital mortality was 15.2% (Table-I).

**Table I:** Demographics, disease severity, disposition, complications and outcomes of patients (n=310).

Characteristics	Results
Age (mean + SD)	57.57 ( ± 14.2)
**Gender (n, %)**	
Male	225 (72.6%)
Female	85 (27.4%)
**Disease Severity *(n, %)***	
Mild	57 (18.4%)
Moderate	92 (29.7%)
Severe to critically ill	161 (51.9%)
**Disposition from ER *(n, %)***	
General ward	91 (29.4%)
Monitored bed	171 (55.2%)
Intensive care unit	48 (15.5%)
**Complications *(n, %)***	
Acute respiratory distress syndrome	115 (37.1%)
Myocardial injury	33 (10.7%)
Deep vein thrombosis	2 (0.6%)
Pulmonary Embolism	1 (0.3%)
**Outcomes *(n, %)***	
Alive	263 (84.8%)
Expire	47 (15.2%)

Biomarkers like CRP, PCT, Ferritin, LDH, and D- dimer were performed in all included patients. Regarding complications, the median value with interquartile range was calculated for each biomarker. Patients who initially had or later developed ARDS had significantly higher values of all biomarkers (P <0.05). Similarly, those patients who had MI during hospitalization had significantly higher values of LDH and D-dimer (P <0.05). None of the biomarkers was significantly elevated in patients who developed DVT and PE (P >0.05) Table-II. All patients who couldn’t survive had significantly higher median values of all biomarkers (P <0.05) Table-III.

**Table II:** Comparison of different biomarkers significant difference among complications of COVID-19 disease.

Complications	CRP	PCT	Ferritin	LDH	D-dimer
**ARDS**					
Yes	155.78 [216.92- 77]	0.22 [0.7- 0.12]	999.9 [1771.1- 406]	564 [733- 429]	1.9 [4.8- 0.8]
No	71.36 [172.73- 26.75]	0.14 [0.3- 0.07]	544 [1167- 290]	411 [510- 339]	1 [1.9- 0.5]
P-value	<0.001*	<0.001*	<0.001*	<0.001*	<0.001*
**Acute_MI**					
Yes	158.81 [195.97- 77]	0.53 [1.05- 0.24]	651.3 [1285- 343.5]	532 [768- 414]	3 [6.7- 1]
No	102.18 [184.78- 34.05]	0.15 [0.31- 0.08]	674.95 [1310.05- 329.85]	449 [578- 351.5]	1.1 [2.3- 0.55]
P-value	0.063	<0.065	0.779	0.008*	<0.001*
**DVT**					
Yes	145.85 [185- 106.7]	2.02 [3.6- 0.44]	2598.15 [5078- 118.3]	746.5 [1042- 451]	11.15 [22- 0.3]
No	106.43 [187.77- 36.55]	0.18 [0.4- 0.09]	674.95 [1293.3- 338.85]	452 [604.5- 359]	1.1 [2.9- 0.6]
P-value	0.558	0.079	0.912	0.246	0.843
**Acute_PE**					
Yes	102 [102- 102]	0.38 [0.38- 0.38]	843 [843- 843]	569 [569- 569]	2 [2- 2]
No	107 [187.67- 37]	0.18 [0.4- 0.09]	669 [1294- 337.6]	451 [607- 359]	1.1 [2.9- 0.6]
P-value	0.96	0.431	0.828	0.454	0.542

**Table III:** Baseline Clinical Biomarkers Stratified by Survival vs Non-Survival.

Characteristics	Survival Outcome		**p-value

	Survival	Non-Survival	
Total	263 (84.8%)	47 (40.1%)	-
**Clinical characteristics [Median (IQR)]**			
CRP	96.85[34-183]	157[77-216.92]	0.002*
PCT	0.15[0.08-0.38]	0.28[0.15-1.05]	<0.001*
Ferritin	610[326.2-1247]	1120[420.3-2573]	0.003*
LDH	434[352-533]	630[414-770]	<0.001*
D-dimer	1[0.5-2.2]	3.5[1.4-10]	<0.001*

In univariate analysis, it was observed that increased values of all biomarkers were significantly associated with the prediction of in-hospital mortality using binary logistic regression analysis (OR > 1.0, P <0.05). In multivariate analysis only two biomarkers i.e., LDH and D-dimer at admission were significantly associated with increased odds of mortality (P <0.05) Table-IV.

**Table IV:** Univariate and Multivariate Analysis using Logistic-Regression: Analysis for in-hospital mortality.

Biomarkers	Univariate Analysis		Multivariate Analysis	

	Sig.	OR [95% C.I]	Sig.	OR [95% C.I]
CRP	0.002	1.12 [1.00 - 1.52]	0.067	0.99 [0.97 -1.23]
PCT	0.028	1.08[1.00 - 1.16]	0.128	1.05 [0.98 -1.12]
Ferritin	0.035	1.00[1.00 - 1.00]	0.061	0.99 [0.98 -1.00]
LDH	<0.001	1.48 [1.21 - 2.87]	0.001	1.32 [1.12 -1.64]
D-dimer	<0.001	1.80 [1.51 - 4.19]	0.004	1.54 [1.26 -3.86]

**Fig.1:**
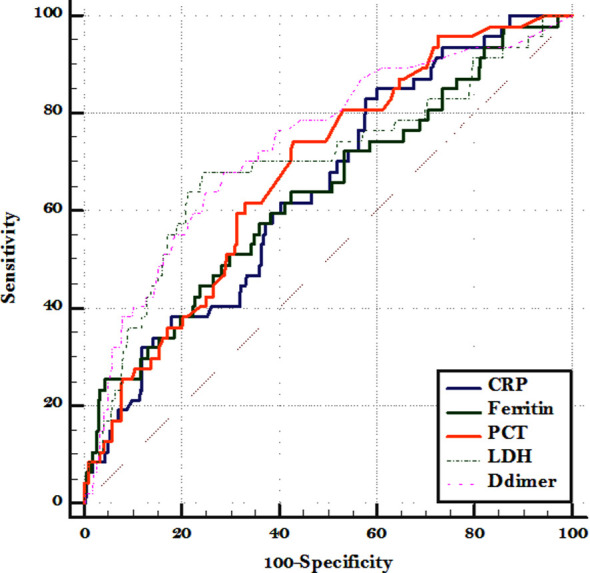
Receiver operating curve for in-hospital mortality with different biomarkers.

The cuff-off values of all the biomarkers were analyzed to evaluate the discrimination validity. Employed the ROC curve and found the area under the curve of each biomarker. The sensitivity and specificity were found to be stable. As indicated in Fig.1 the area under curve (AUC=0.73) implied a perfect accuracy of serum D-dimer level of >1.8 in COVID-19 patients as a predictor of in-hospital mortality with sensitivity and specificity of 68.09% and 71.1%. Similarly, the AUC = 0.69 of serum LDH >537 showed moderate sensitivity 68.09% but highest specificity 75.67% Table-V.

**Table V:** Sensitivity and specificity analysis at optimal cut off of various biomarkers for in-hospital mortality.

Biomarkers	AUC [95% C.I]	Criterion	Sensitivity [95% C.I]	Specificity [95% C.I]	+LR [95% C.I]
CRP	0.643 [0.562 to 0.725]	>67	82.98 [69.2 - 92.4]	42.21 [36.2 - 48.4]	1.44 [1.2 - 1.7]
Ferritin	0.636 [0.545 to 0.727]	>990.8	57.45 [42.2 - 71.7]	63.88 [57.8 - 69.7]	1.59 [1.2 - 2.1]
PCT	0.679 [0.601 to 0.757]	>0.194	74.47 [59.7 - 86.1]	57.03 [50.8 - 63.1]	1.73 [1.4 - 2.2]
LDH	0.695 [0.603 to 0.788]	>537	68.09 [52.9 - 80.9]	75.67 [70.0 - 80.7]	2.8 [2.1 - 3.7]
D-dimer	0.735 [0.654 to 0.817]	>1.8	68.09 [52.9 - 80.9]	71.1 [65.2 - 76.5]	2.36 [1.8 - 3.1]

## DISCUSSION

The study highlights the importance of biomarkers in predicting complications and in-hospital mortality in patients diagnosed with COVID-19. In this study, more than half of the patients had severe to critical disease at presentation to the ED and around 37% of them developed complications such as ARDS, MI, DVT, and PE, and almost 15% of the patients expired during the hospital stay. This study shows that in patients with COVID-19 who were diagnosed or later developed ARDS, the median values of all biomarkers were elevated whereas in patients who developed MI during the hospital stay the median values of LDH and D-dimer were significantly elevated. We also demonstrated that elevated levels of LDH and D-dimer upon admission had a strong association with increased odds of in-hospital mortality.

In COVID-19 there is a systemic inflammatory phase in which inflammatory biomarkers, such as CRP, ferritin, PCT, D-dimer, and LDH are markedly elevated. This phase of hyperinflammation is mediated by increased levels of cytokines that cause a cascade of the severe inflammatory process throughout the body and can lead to complications like ARDS, multi-organ failure, and cardiopulmonary collapse.^8^,^9^ This study explored the relationship between these biomarkers and complications of COVID-19 and found that patients with high median values of CRP, ferritin, LDH, D-dimer, and procalcitonin were prone to develop the severe form of disease and development of ARDS. The results are similar with other studies that positively correlate elevated levels of laboratory biomarkers with disease severity and respiratory failure requiring invasive or noninvasive mechanical ventilation, with a nearly five-fold increased risk of developing ARDS.^10^-^15^

There is some evidence that COVID-19 may cause a hypercoaguable state resulting in severe complications like Acute Pulmonary Embolism (PE) and Acute Myocardial Infarction (MI). The frequency of myocardial injury (as reflected by elevation in cardiac troponin levels) is variable among hospitalized patients with COVID-19, with reported frequencies of 7 to 28 percent.^16^ It may be due to direct myocardial injury from hemodynamic instability or hypoxemia, stress cardiomyopathy, inflammatory myocarditis, or thrombosis due to hypercoagulable condition. Systemic inflammation can lead to cytokine storm which may destabilize the plaques of the coronary artery and lead to myocardial infarction.^17^ In this study, we found that patients with an acute myocardial injury had significantly elevated serum levels of LDH and D-dimer. Our results are in alignment with a study conducted by Berger, Jeffrey S., et al. which showed that COVID patients who developed myocardial injury had increased levels of D-dimer at presentation.^18^

Predicting mortality from initial levels of biomarkers can be advantageous. It allows us to stratify patients earlier so that appropriate interventions can be facilitated. Furthermore, in resource-limiting settings, laboratory tests that predict outcomes can be used to support decisions regarding the escalation of medical care. This study shows that the median levels of all biomarkers were significantly elevated in patients who got expired. In univariate analysis increased levels of all biomarkers were significantly associated with increased odds of mortality. Furthermore, in multivariate analysis increased levels of LDH and D-dimer were significantly associated with increased odds of mortality. Our results are consistent with other studies that showed a positive association between high levels of these biomarkers and mortality.^19^-^21^

### Limitations of the study:

This is a single-center retrospective study hence the results cannot be generalized to other populations. Further studies are required to validate these results. Another limitation is that we were unable to monitor the trend of the biomarkers level during the course of disease, which can significantly affect the clinical course and ultimately the outcomes of the disease.

## CONCLUSION

Serum CRP, ferritin, procalcitonin, LDH, and D-dimer levels at the time of admission can predict complications like ARDS and MI and also predict mortality in COVID-19 infection. Serum LDH and D-dimer are the best amongst them for predicting mortality. Availability of these biomarkers in emergency rooms can help the healthcare providers to predict the probable complications in these patients thus allowing for early and appropriate allocation of healthcare resources.
